# Appraisal of the Possible Role of PPAR*γ* Upregulation by CLA of Probiotic *Pediococcus pentosaceus* GS4 in Colon Cancer Mitigation

**DOI:** 10.1155/2023/9458308

**Published:** 2023-02-24

**Authors:** Vinay Dubey, Alok Kumar Mishra, Asit Ranjan Ghosh

**Affiliations:** ^1^Microbial Molecular Biology Laboratory, Department of Integrative Biology, School of Bio Sciences and Technology, Vellore Institute of Technology, Vellore, Tamil Nadu 632014, India; ^2^Department of Microbiology, School of Bioengineering and Biosciences, Lovely Professional University, Phagwara, Punjab, India

## Abstract

The prevalence of colon cancer (CC) is increasing at the endemic scale, which is accompanied by subsequent morbidity and mortality. Although there have been noteworthy achievements in the therapeutic strategies in recent years, the treatment of patients with CC remains a formidable task. The current study focused on to study role of biohydrogenation-derived conjugated linoleic acid (CLA) of probiotic *Pediococcus pentosaceus* GS4 (CLA_GS4_) against CC, which induced peroxisome proliferator-activated receptor gamma (PPAR*γ*) expression in human CC HCT-116 cells. Pre-treatment with PPAR*γ* antagonist bisphenol A diglycidyl ether has significantly reduced the inhibitory efficacy of enhanced cell viability of HCT-116 cells, suggesting the PPAR*γ*-dependent cell death. The cancer cells treated with CLA/CLA_GS4_ demonstrated the reduced level of Prostaglandin E2 PGE_2_ in association with reduced COX-2 and 5-LOX expressions. Moreover, these consequences were found to be associated with PPAR*γ*-dependent. Furthermore, delineation of mitochondrial dependent apoptosis with the help of molecular docking LigPlot analysis showed that CLA can bind with hexokinase-II (hHK-II) (highly expressed in cancer cells) and that this association underlies voltage dependent anionic channel to open, thereby causing mitochondrial membrane depolarization, a condition that initiates intrinsic apoptotic events. Apoptosis was further confirmed by annexin V staining and elevation of caspase 1p10 expression. Taken all together, it is deduced that, mechanistically, the upregulation of PPAR*γ* by CLA_GS4_ of *P*. *pentosaceus* GS4 can alter cancer cell metabolism in association with triggering apoptosis in CC.

## 1. Introduction

Colon cancer (CC) is increasing to a pandemic scale and is accompanied by subsequent morbidity and mortality [1]. The etiology of CC is multifactorial including acquisition of mutation, consecutive immune response, gut microbiome disbalance or leaky gut, and cellular metabolic alterations. These factors may lead to oncogenic transformation of colonocytes, leading to the CC progression. Therefore, regulation of key transcriptional regulators associated with metabolic pathways becomes promising and selective.

The peroxisome proliferator-activated receptor gamma (PPAR*γ*), a cluster of nuclear transcription factor, regulates a wide array of cellular metabolic events including lipid metabolism, insulin sensitivity, cell proliferation, and inflammatory regulatory signaling [2]. Among its three different isoforms, PPAR*γ* 2 is widely expressed in lower part of distal gut (colon), adipose tissue, and macrophages [2]. Upon activation by wide variety of ligands, such as polyunsaturated fatty acids (i.e., arachidonic acid, 9, 11- and 10, 12-conjugated linoleic acid (CLA), 15-deoxy-delta12-14-PGJ2, and 13-HODE) and thiazolidinediones, PPAR*γ* modulates DNA transcription by binding to peroxisome proliferator responsive elements, which consequently disrupt the metabolic events of cancer cells *via* inhibition of Akt-driven oncogenic signalling, restriction of cell proliferation, and reduction in transcriptional activation of pro-inflammatory transcription factor, leading to forced metabolic destruction of cancer cells [3]. The PPAR*γ* role in cancer suppression is unique and intricate. The various targets have been investigated during the investigation of anti-carcinogenic effects of PPAR*γ* agonists. The prevention of E2F/DP binding, p21 stimulation, or suppression of cyclin D1 have been associated with the control of cellular proliferation by PPAR*γ* [4]. CC HT-29 cells were observed with lowered expression of Cyclin Dependent Kinase (CDK) proteins when treated with ciglitazone [5]. Later, it was found that PPAR*γ* transactivates the CDK inhibitors p 2 1C I P and p 2 7K I P 1, which consequently inhibit CDK binding to cyclin, and thus, cell cycle is arrested [6]. Furthermore, PPAR*γ* also inhibits cyclin E1 restricted cancer cell progression by inhibiting their entry at G1/S phase in CC cells [7]. Previous studies have demonstrated that PPAR*γ* agonist rosiglitazone (BRL4653) can restrict cellular growth through upregulation of p53, whereas this response was no longer seen in the presence of PPAR*γ* antagonist GW9662. Moreover, BRL also induced p53 effector p21 (WAF1/Cip1) in breast cancer MCF7 cells resulting into apoptosis [8]. Thus, modulating the PPAR*γ* in colon becomes a potential drug target that can be exploited for the treatment of CC.

Recently, we revealed that CLA produced by probiotic *Pediococcus pentosaceus* GS4 (CLA_GS4_) has a strong antiproliferative and protective efficacy against CC [9]. CLA a natural ligand for PPAR*γ* forms a nuclear complex and activates transactivation, which subsequently forms the inhibitory complex with p65 subunit of Nuclear factor kappa B (NF-*κ*B), leading to the inhibition of proinflammatory and proliferation signalling. Furthermore, PPAR*γ* induces consequences in mitochondria mediated cell metabolism, generating oxidative stress and electron flux, which favours the apoptotic cascades [10]. Based on these observations, we further investigated the possible role of PPAR*γ* upregulation by CLA of probiotic *P. pentosaceus* GS4 in CC mitigation.

## 2. Materials and Methods

The probiotic *Pediococcus pentosaceus* GS4 MTCC 12683 (Genbank accession no: HMO44322) was cultured in De Mann Rogosa Sharpe broth/agar at 37°C following standard microbiological procedures. CLA_GS4_ was prepared as reported elsewhere [11]. The human CC cell line HCT-116, which was procured from the National Centre for Cell Science, Pune, India, and was maintained in Dulbecco's Modified Eagle Medium (DMEM) supplemented with 10% (v/v) heat inactivated Fetal bovine serum (FBS) and 100 *μ*g/mL of streptomycin and 10 U/mL of penicillin at 37°C in 5% CO_2_ atmosphere (CO_2_ incubator, Sanyo, Osaka, Japan). The CLA (mixture of *trans*-10, *cis*-12 and *cis*-9, *trans*-11 CLA), linoleic acid, and bisphenol A diglycidyl ether (BADGE) were purchased from Sigma (St. Louis, MO, USA). The acridine orange (AO), ethidium bromide (EtBr), propidium iodide (PI), 4′,6-diamidino-2-phenylindole, 2′,7′-dichlorofluorescin diacetate, and Rhodamine-123 (Rh123) were cell culture grade purchased from HiMedia, Mumbai, India. Primary antibodies against PPAR*γ* (CST 2435), 5-LOX (3289), NF-*κ*B (8242), p53 (2524), p21^WAF^ (37543), Bax (2772), Bcl_2_ (15071), cleaved Poly (ADP-ribose) polymerase (PARP) (5625), caspase 3 (9662), and glyceraldehyde-3-phosphate dehydrogenase (GAPDH) (5174) were purchased from Cell Signaling Technology (Beverly, MA, USA). The COX-2 primary antibody was obtained from TaKaRa, Shiga, Japan. The Pierce TMB-Blotting 1-step solution (34018) was procured from Thermo Fisher, Massachusetts, USA.

### 2.1. Kits

The various commercial kits were brought from different companies. The annexin V-Fluorescein isothiocyanate (FITC) apoptosis kit (K101) was procured from BioVision, Waltham, MA, USA. Prostaglandin E_2_ (PGE_2_) ELISA kit (514010), glucose uptake (600470), and Nicotinamide adenine dinucleotide/Nicotinamide adenine dinucleotide (Hydrogen) (*NAD/NADH*) cell-based assay kits (600480) were purchased from Cayman, USA. Human IL-8 standard TMB ELISA kit (900-T18) was purchased from PeproTech Asia, Rehovot, Israel.

### 2.2. Molecular Docking and LigPlot Analysis

The Pubchem database was used to acquire the SMILES of the ligands (c9-, t11-CLA, and BADGE). The 3D structure of the ligands was obtained through online tool of NCI/CADD group by uploading SMILES files of ligands. These files were used further for molecular docking analysis and interaction studies. The molecular docking of PPAR*γ* protein (PDB ID: 3NOA) with the ligands (BADGE and 9, 11-CLA) and between 9, 11-CLA and hexokinase-II (hHK-II) (HK-II; PDB ID: 2nzt) was performed using AutoDock (4.0) based on the Lamarckian genetic algorithm (Hu and Shelver, 2003). The active site was defined using AutoGrid. The grid size was set to 90 × 90 × 90 points with a grid spacing of 0.375 Å, centred on the binding site of the protein molecules [12]. The grid box included the entire protein molecule to ensure enough space for the ligand translational and rotational walk. 50° for rotation and step sizes of 1 Å for translation were chosen. The maximum number of energy evaluations was set to 25,000,000, and 100 runs were performed for each ligand. The genetic algorithm was set to generate 27,000 operations on a single population of 200 individuals for each of the 100 independent runs. Default values were used for crossover, mutation, and elitism operator weights (0.80, 0.02, and 1.00, respectively). Docked conformations of 100 runs were clustered on the basis of their root mean square (RMS) deviation tolerance of the ligand conformation [13]. Consequently, the best docked conformation was selected and visualized using PyMOL 0.99. The best among top 10 binding was analysed for interaction using LigPlot, which elucidate 2D schematic representation of all the bonds formed during ligand and protein or receptor (amino acid residues) binding [14].

### 2.3. BADGE Treatment

**Figure 1:**
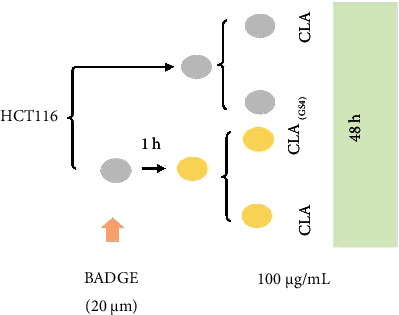
Schematic of treatment schedule.

A stock solution (20 mM) of BADGE was prepared in Dimethyl sulfoxide (DMSO). The HCT-116 cells were pre-treated with 20 *μ*M BADGE in serum free DMEM for 1 hour followed by CLA or CLA (GS4) treatment for 48 hours, as depicted in Figure 1.

### 2.4. Cell Viability Assay

The HCT-116 cells (5.0 × 10^3^ cells/well) were seeded in 96-well plates for overnight. The cells were pre-treated with BADGE (20 *μ*M) for 1 hour, followed by CLA/CLA_GS4_ (100 *μ*g/mL) treatment for 48 hours. The cytotoxicity was determined using 3-[4,5-dimethylthiazol-2-yl]-2,5 diphenyl tetrazolium bromide (MTT) assay, and absorbance was recorded using a microplate reader (Bio-Rad 680, California, USA), and data were presented as percent of cell viability.

### 2.5. The Cell Morphological Analysis and AO/EtBr Staining

The HCT-116 cells (2.0 × 10^4^ cells/well) were seeded in 24-well plate and given treatment as described earlier above. The cell morphology was examined under bright-field microscope (OlympusIX70; Olympus, Tokyo, Japan) and photographed in 10× magnification. The cells were stained with fluorescent dye AO/EtBr as described earlier [15].

### 2.6. Mitochondrial Membrane Potential

After scheduled treatment for 24 hours, the HCT-116 cells (5.0 × 10^3^ cells/well) were stained with 10 *μ*M Rh123 dye for 30 minutes in dark condition. The fluorescence intensity was determined by excitation at 511 nm and emission at 534 nm, and mitochondrial membrane potential (MMP) (*Ψ*_m_) was calculated and expressed as % of control.

### 2.7. FACS

After scheduled treatment, the HCT-116 cells were harvested and rinsed with sterile Phosphate-buffered saline (PBS) twice and fixed using 70% ethanol and stained with PI for 30 minutes in dark condition, and the intensity was measured by flow cytometry (50,000 cells for each sample) (Riccardi and Nicoletti, 2006). For annexin V staining, the cells were stained using annexin V-FITC apoptosis detection kit (BioVision). Briefly, following scheduled treatment, the cells were stained with annexin V-FITC and PI at room temperature in dark condition. The stained cells (50,000 cells/sample) were analysed by flow cytometry (Ex = 488 nm; Em = 530 nm) using FITC signal detector FL1 and PI staining FL2. The acquired data were analysed using the cyflogic software.

### 2.8. Glucose Uptake Assay

The 2-(N-(7-Nitrobenz-2-oxa-1,3-diazol-4-yl)Amino)-2-Deoxyglucose (2-NBDG) cell-based glucose uptake assay was performed. Briefly, following pre-treatment with BADGE (20 *μ*M) for 1 hour, the cells were treated with 100 *μ*g/mL of CLA/CLA_GS4_ in glucose free DMEM for 48 hours. Thirty minutes before the end of the treatment, 2-NBDG was added to a final concentration of 100 *μ*g/mL in glucose free medium. The cells were harvested, and assay was performed as according to the manufacture's guidelines (Cayman Chemical, Michigan, USA). The fluorescence was recorded using fluorospectrometric with fluorescent filter (excitation/emission = 485 nm/535 nm). The results were expressed as glucose uptake (% of control).

### 2.9. NAD^+^/NADH Estimation

The NAD^+^/NADH was estimated using NAD^+^/NADH cell-based assay kit according to manufacturer's protocol (Cayman Chemical). The NAD^+^ concentration in samples was calculated using NAD^+^ standard curve and expressed as NAD^+^ (nM).

### 2.10. PGE_2_ Estimation

After scheduled treatment, the cell culture supernatant was subjected to competitive ELISA using PGE_2_ ELISA kit (Cayman Chemical).

### 2.11. Statistical Analysis

The experiments were performed in triplicate, and the data were expressed as means ± SE. One-way Analysis of Variance (ANOVA) was used for the evaluation of differences in means. The analysis was performed using the GraphPad Prism version 5.0 (GraphPad software) and the SPSS.14 software (IBM Corporation, Armonk, NY, USA). MATLAB (R2013B, The Mathworks, Inc., Natick, MA, USA) was used in plotting the 3D surface plot graph and the correlation study. Statistical analysis among means of control and experimental groups was performed using the Newman–Keuls multiple comparison test at a significance level of *P* ≤ 0.05.

## 3. Results

### 3.1. Dynamic Study of CLA Interaction with PPAR*γ*

**Figure 2:**
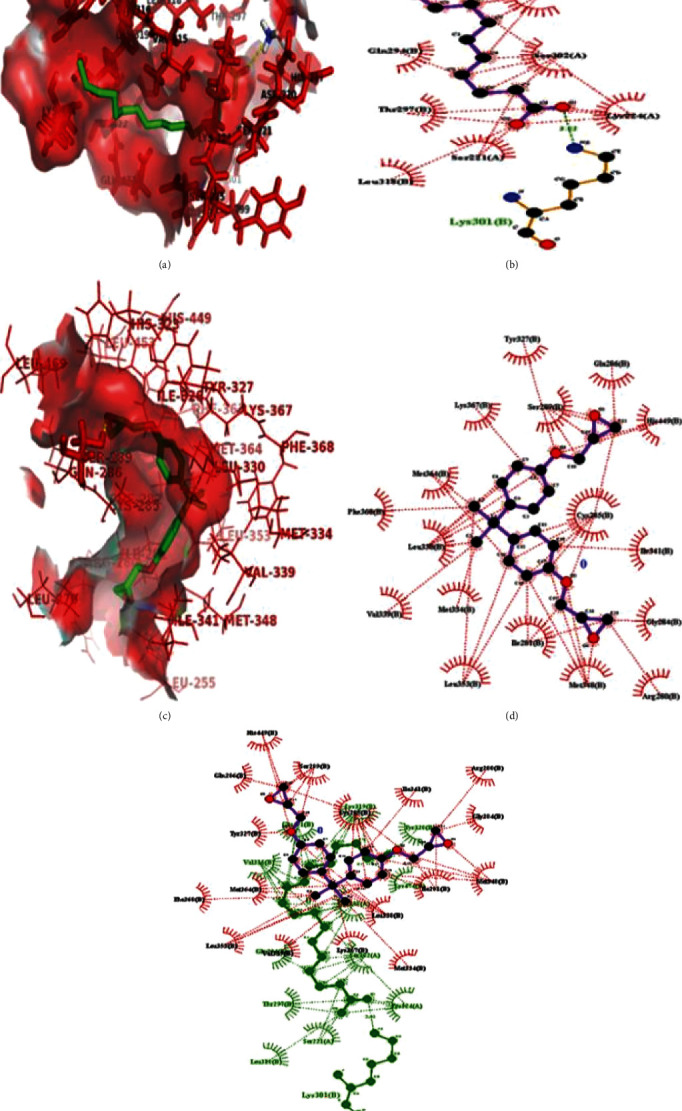
The c9t11-CLA and BADGE can bind to human PPAR*γ*: The hPPAR*γ* protein was retrieved from protein data bank and molecular docking was performed using AutoDock followed by LigPlot analysis. Binding energy was used in order to rank structures in each cluster. (a) PyMOL view of molecular interaction between c9t11-CLA and hPPAR*γ*. (b) 2D docking complex of c9t11-CLA with the amino acid residues of hPPAR*γ*. (c) PyMOL view of molecular interaction between BADGE and hPPAR*γ*. (d) 2D docking complex of BADGE with the amino acid residues of hPPAR*γ*. (e) The merge view of 2D docking complex of c9t11-CLA and BADGE with the amino acid residues of hPPAR*γ* protein.

The dynamic interaction studies using bioinformatic tools suggested that ligand c9t11CLA binds with PPAR*γ* (PDB ID: 3NOA; binding energy [BE]: −5.02 kcal/mol; Figure 2(a)) and interacts with active site residues of hPPAR*γ* protein, such as LEU318(B), SER221(A), THR297(B), GLN294(B), VAL315(B), GLU471(B), LYS319(B), TYR320(B), LYS474(B), GLN298(A), SER302(A), and LYS224(A), through hydrophobic interactions. Furthermore, docking studies were performed using AutoDock (4.0; Figure 2(b)). The possible ligand binding site of c9t11CLA in hPPAR*γ* was derived from molecular docking technique by generating 100 structures and setting a RMS tolerance at 4 Å to form cluster. BE was used to rank structures belonging to each cluster. Based on lowest BE and higher number of conformations in given cluster, best docked conformation was selected. It was observed that c9t11CLA displayed efficient binding with hPPAR*γ* (PDB ID: 3NOA; BE: −5.02 kcal/mol). Moreover, BADGE an antagonist of PPAR*γ* also showed efficient binding to hPPAR*γ* (BE: −6.46 kcal/mol; Figure 2(c)). Interestingly, c9t11-CLA and BADGE do not share any amino acids of active site residues of PPAR*γ* (Figures 2(d) and 2(e)).

### 3.2. Biohydrogenation-Derived CLA of Probiotic *P. pentosaceus* GS4 Induced PPAR*γ* Expression in Human CC Cells

**Figure 3:**
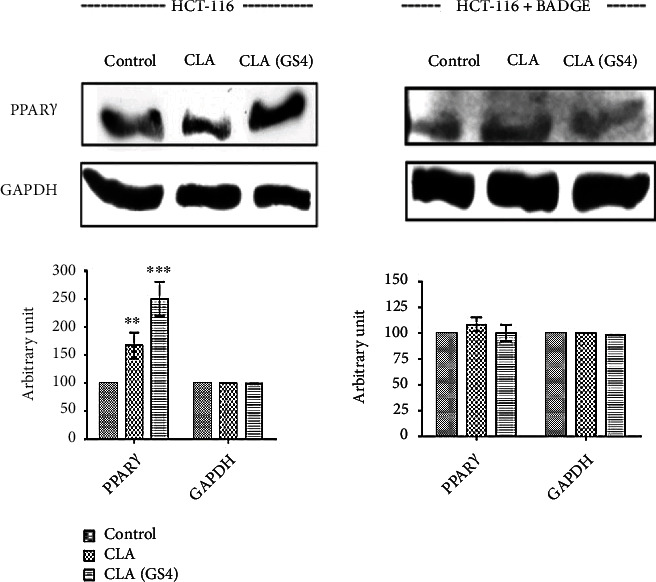
Biohydrogenation-derived CLA of probiotic GS4 induced PPAR*γ* in HCT-116 cells: The HCT-116 cells were pre-incubated for 1 hour with/without BADGE (20 *μ*M) before treatment of CLA/CLA_GS4_ for 48 hour. The PPAR*γ* expression was examined by western blot analysis. The GAPDH was used as loading control. The densitometric analysis of bands was evaluated using the ImageJ software. Data (means ± SE) were statistical analysed by one way-ANOVA with *post hoc* Newman–Keuls analysis and depicted as ∗*versus* respective control.

Since CLA is a natural ligand of PPAR*γ*, the effect of CLA_GS4_ on PPAR*γ* was examined using western blot. The CLA/CLA_GS4_ induced PPAR*γ* expression in HCT-116 cells; however, this effect abolished when cells were pre-treated with BADGE (Figure 3). It is inferred from the observations derived from both *in silico* and western blot analysis that BADGE pre-treatment induced conformational changes in hPPAR*γ*, making unavailable for CLA binding, thereby no significant change in PPAR*γ* expression in HCT-116 cells.

### 3.3. The Probiotic *P. pentosaceus* GS4 Inhibited PPAR*γ*-Dependent COX-2 and 5-LOX Expression

**Figure 4:**
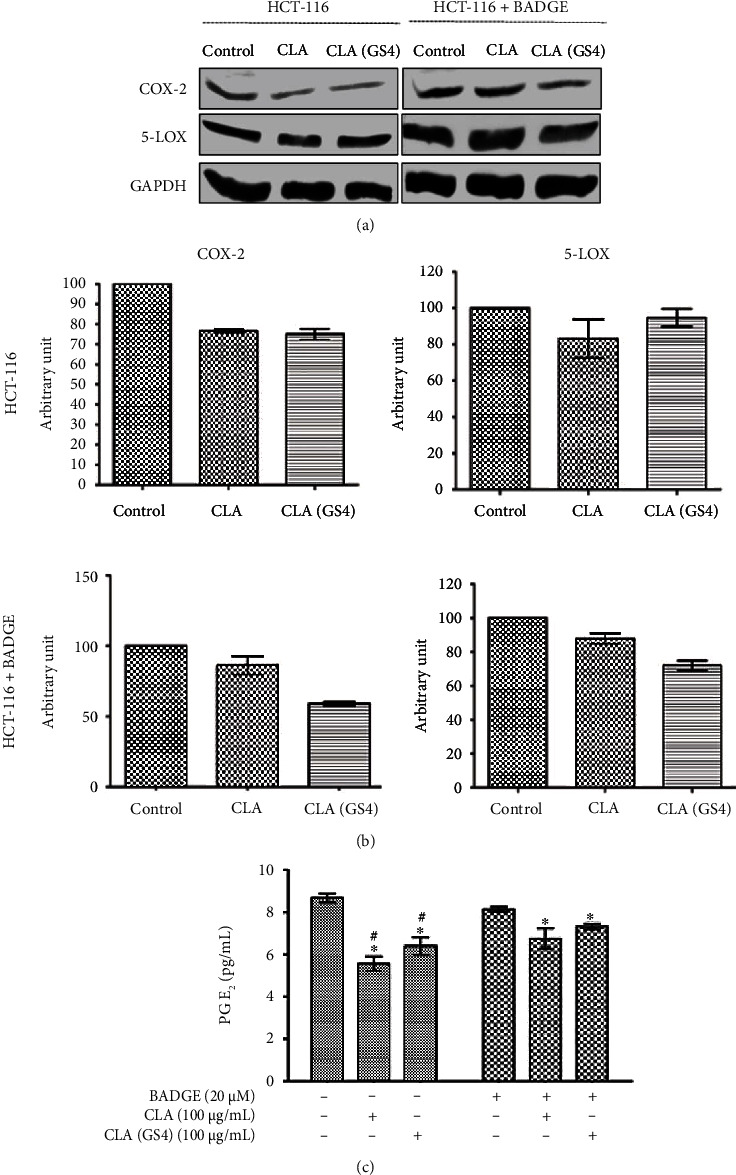
The biohydrogenation-derived CLA altered down effectors of PPAR*γ* in HCT-116. The HCT-116 cells were pre-incubated for 1 hour with/without BADGE (20 *μ*M) before treatment of CLA/CLA_GS4_ for 48 hour. (a) The COX-2 and 5-LOX expression were studied by western blot analysis. GAPDH was used as a loading control. (b) The densitometric analysis of bands was evaluated using the ImageJ software version 1.49. (c) The PGE_2_ level in HCT-116 cells. Data (means ± SE) and were statistical analysed (*P* ≤ 0.05) by one way-ANOVA with *post hoc* Newman–Keuls analysis and depicted as ∗*versus* control; #*versus* BADGE control.

Many studies demonstrated the COX-2 and 5-LOX role in tumorigenesis, and selective inhibition of COX-2 efficiently prevented the experimental CC and induced apoptosis. Furthermore, CLA is known to inhibit COX-2 expression and its metabolic-derived product PGE_2_ level in mammary tumors [16]. Therefore, the efficacy of CLA_GS4_ in the suppression of COX-2, 5-LOX, and PGE_2_ was evaluated. The CLA/CLA_GS4_ treatment significantly (*P* ≤ 0.05) reduced the COX-2 expression (Figures 4(a) and 4(b)). Interestingly, this efficacy was eliminated when the cells were pre-treated with BADGE (20 *μ*M) for 1 hour. Concomitantly, CLA/CLA_GS4_ lowered the secreted PGE_2_ in a PPAR*γ*-dependent manner. These results indicated that CLA_GS4_ regulates PPAR*γ*-dependent COX-2 and 5-LOX expression, thereby suppressing PGE_2_ secretion (Figure 4(c)).

### 3.4. The Probiotic-Derived CLA Induced PPAR*γ*-Dependent Metabolic Alterations in CC Cells

**Figure 5:**
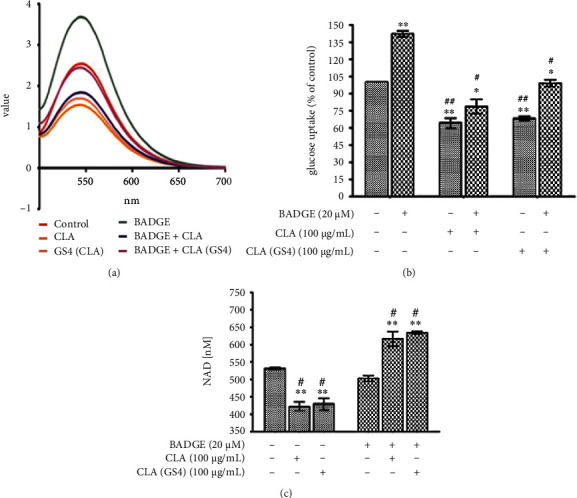
The biohydrogenation-derived CLA of probiotics GS4 altered cell metabolism in HCT-116. The HCT-116 cells were pre-treated with BADGE (20 *μ*M) for 1 hour followed by CLA/CLA_GS4_ treatment for 48 hour treatment. The glucose uptake was estimated by incubation with 100 *μ*g/mL 2-NBDG for 30 minutes followed by estimation of accumulated 2-NBDG using fluorospectrometer. (a) Scanning spectrum. (b) The calculated glucose uptake (% of control) among different groups. (c) The NAD^+^ (nm) was estimated using NAD^+^ assay kit (Cayman Chemical). Statistical analysis of data (means ± SE) was determined by one way-ANOVA with *post hoc* Newman–Keuls analysis and depicted as ∗*versus* control; # *versus* BADGE control.

We examined the effect of PPAR*γ* upregulation on glucose uptake efficiency of cancer cells (Figure 5(a)). The HCT-116 cells were treated with CLA/CLA_GS4_ without glucose and monitored the 2-NBDG uptake. The CLA/CLA_GS4_ significantly (*P* ≤ 0.05) reduced the glucose uptake (Figure 5(b)). Interestingly, PPAR*γ* antagonist BADGE pre-treatment significantly (*P* ≤ 0.05) abolished this inhibitory effect of CLA/CLA_GS4_, which was evident from lower glucose uptake when compared with control. This can be deduced from these observations that CLA can alter PPAR*γ*-dependent glucose consumption efficacy of CC cells.

NAD^+^ plays a pivotal role in producing a reduced product NADH, which has significance in many physiological processes [17]. Moreover, NAD^+^ level appears to have an essential role in fate of cancer cells [18]. To confirm the role of probiotic-derived CLA_GS4_ activated PPAR*γ* role in the regulation of cellular metabolic activity of cancer cells, the current study evaluated cellular NAD^+^ levels in human CC cells. The PPAR*γ*-dependent decreased in the cellular NAD^+^ level was observed in CLA/CLA_GS4_ treatment group when compared with control (Figure 5(c)). Following treatment schedule, the BADGE and control group cells remained non-significantly different. Taken together both glucose uptake and NAD^+^ observation, the study suggests that probiotic CLA_GS4_ inhibited CC, by inducing metabolic collapse.

### 3.5. The Probiotic-Derived CLA Induced PPAR*γ*-Dependent Cell Death in CC Cells

**Figure 6:**
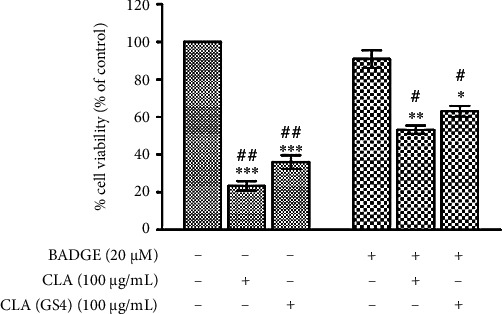
Biohydrogenation-derived CLA of probiotic GS4 induced PPAR*γ*-dependent cell death in HCT-116 cells. The HCT-116 cells were pre-incubated for 1 hour with/without BADGE (20 *μ*M) before treatment of CLA/CLA_GS4_ for 48 hour. The percentage of cell viability was determined by MTT assay. Data (means ± SE) of the % of viability pooled from three independent experiments. Statistical analysis was performed by one way-ANOVA with *post hoc* Newman–Keuls analysis and depicted as ∗*versus* control; #*versus* BADGE control at *P* ≤ 0.05.

The study was extended to nurture the PPAR*γ* dependency in cell death induced by CLA_GS4_, and the HCT-116 cells were pre-treated with a PPAR*γ* antagonist BADGE (20 *μ*M) followed by CLA/CLA_GS4_ treatment. Both CLA and CLA of probiotic GS4 effects were significantly, if not completely, blocked in the presence of 20 *μ*M BADGE (Figure 6), implying that the effects are mediated through the activation of PPAR*γ*. The viability of cells treated BADGE alone did not show any significant difference with control cells. These observations provide convincing evidence that PPAR*γ* activation (at least in part) is required for the antiproliferative effects of biohydrogenation-derived CLA of probiotic GS4 on human CC cells.

### 3.6. The Probiotic-Derived CLA Restricted PPAR*γ*-Dependent Cell Cycle Progression

**Figure 7:**
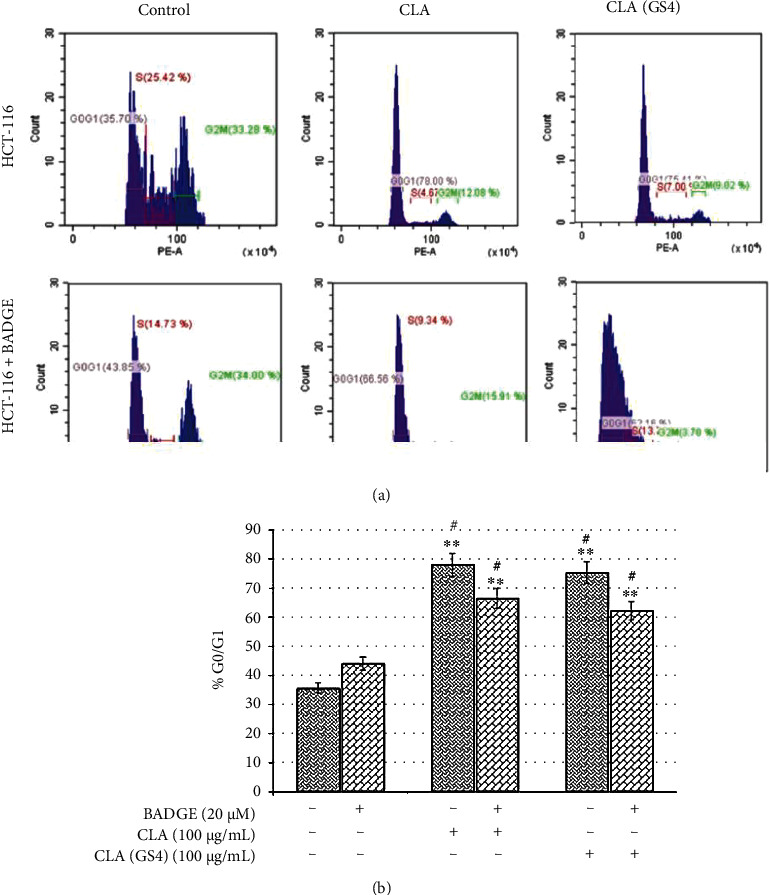
The biohydrogenation-derived CLA of probiotic GS4 arrested cell cycle of HCT-116: The HCT-116 cells were pre-treated with BADGE followed by 100 *μ*g/mL CLA/CLA_GS4_ treatment for 48 hours, subsequently, cells were fixed, and PI staining was performed. The stained cells were analysed by flow cytometry and data were expressed as % of control. Statistical analysis of data (means ± SE) was determined by one way-ANOVA with *post hoc* Newman–Keuls analysis and depicted as ∗*versus* control; #*versus* BADGE control.

Cancer cells were found disrupted cell cycle progression, and its inhibition could induce cell death. The induction of cell death further confirms the anti-proliferative efficacy of probiotic GS4. The cell cycle was analysed using PI staining followed by Fluorescence-activated cell sorting (FACS) analysis.  Figure 7(a) depicted the distribution of cells at different stages of cell cycle. The CLA/CLA_GS4_ treatment arrested cells at G0/G1 stage apparent from higher %cells found at this stage. Though there was reduction in %cells arrested in G0/G1 stage of cell cycle, however, it was found non-significantly different when cells pre-treated with BADGE for 1 hour before CLA/CLA_GS4_ treatment (Figure 7(b)). These observations suggested the PPAR*γ*-dependent (at least in part) in the restriction of cell proliferation mediated by biohydrogenation-derived CLA of GS4.

### 3.7. The Probiotic-Derived CLA Induced PPAR*γ*-Dependent Apoptosis in CC Cells

To further study the role of PPAR*γ* upregulation in probiotic-derived CLA mediated cell death, the apoptosis was analysed at two levels, first at morphological assessment of apoptosis using fluorescent stains (AO/EtBr) and second using FACS-based annexin V staining method.

#### 3.7.1. Morphological Assessment for Apoptosis

**Figure 8:**
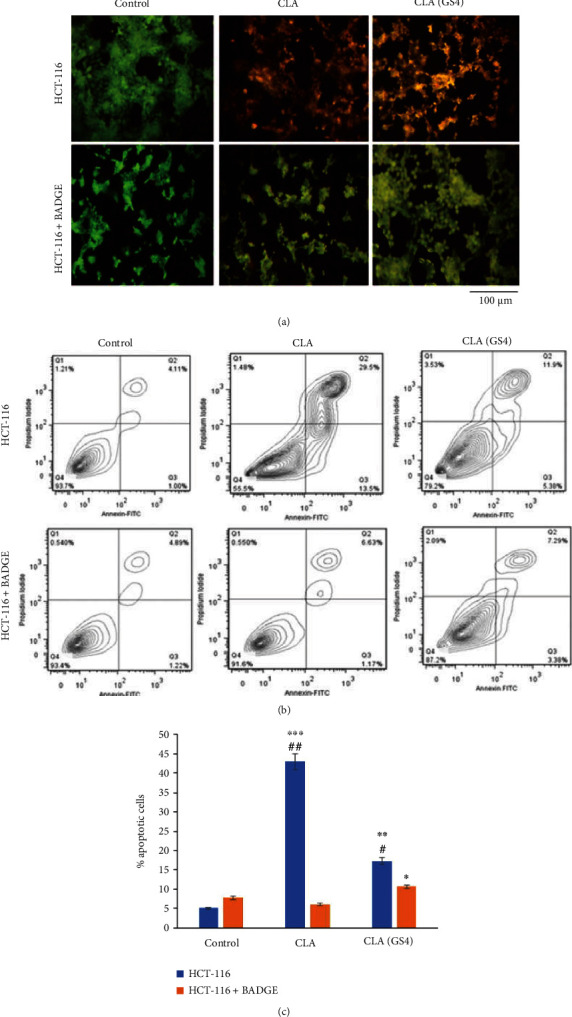
Probiotic *P. pentosaceus* GS4 induced apoptosis in human colon cancer cells: (a) The morphological assessment of apoptosis: The HCT-116 cells were pre-incubated for 1 hour with/without BADGE (20 *μ*M) before treatment with CLA/CLA_GS4_ for 48 hour. Following incubation, the apoptosis was determined at morphological level by staining the cells with dual stain (AO/EtBr) and subsequently images were observed under fluorescence microscope and images were captured (scale 100 *μ*m**)**. FACS based assessment of apoptosis: (b) Following scheduled treatment for 48 hours, the HCT-116 cells were analysed by annexin V/PI staining subsequently FACS analysis. (b) The histogram of percentage of apoptosis at different stage. (c) The graphical representation of % apoptotic cells (apoptotic index: AI, annexin V positive cells) among different groups. Statistical significance was indicated as ∗*versus* control; #*versus* BADGE control.

The apoptotic characteristics at morphological level were determined by dual fluorescent dye AO/EtBr staining technique. CLA/CLA_GS4_ treatment increased the green orange fluorescence indicating that chromatin condensation and nuclear fragmentation have occurred, thereby incorporating EtBr (late apoptotic stage; [13]; Figure 8(a)). However, pre-treatment of PPAR*γ* antagonist rescued cells as apparent from reduced incorporation of EtBr; thus, cells were light orange-green florescent with intact nuclei. In contrast, the live control cells showed green colour florescence during florescence microscope observation.

#### 3.7.2. Flow Cytometric-Based Assessment for Apoptosis

The flow cytometric analysis represented the quantitative data of apoptosis upon treatment of cancer cells with CLA/CLA_GS4_. As shown in histogram (Figure 8(b)), after 48 hours of treatment, the apoptotic index (AI; annexin V positive stained cells) was higher when compared with control cells, whereas the annexin V positive stained cells or AI were significantly reduced when PPAR*γ* was suppressed by BADGE, suggesting the PPAR*γ* role in triggering apoptosis (Figure 8(c)). Taken together both morphological and flow cytometric-based observations, it can be inferred that biohydrogenation-derived CLA of GS4 can induce PPAR*γ*-dependent apoptosis in CC cells.

### 3.8. The Probiotic-Derived CLA Induced Mitochondrial Membrane Depolarization in CC Cells

Our observations showed the anti-proliferation is associated with apoptosis; therefore, we further analysed the MMP to nurture the possible role of this specific strain of probiotic in mitochondrial depolarization. The strategy was divided into two phases: first, study of CLA role in mitochondrial membrane using bioinformatic tools and, second, examination of MMP.

#### 3.8.1. CLA Can Bind to HK-II

**Figure 9:**
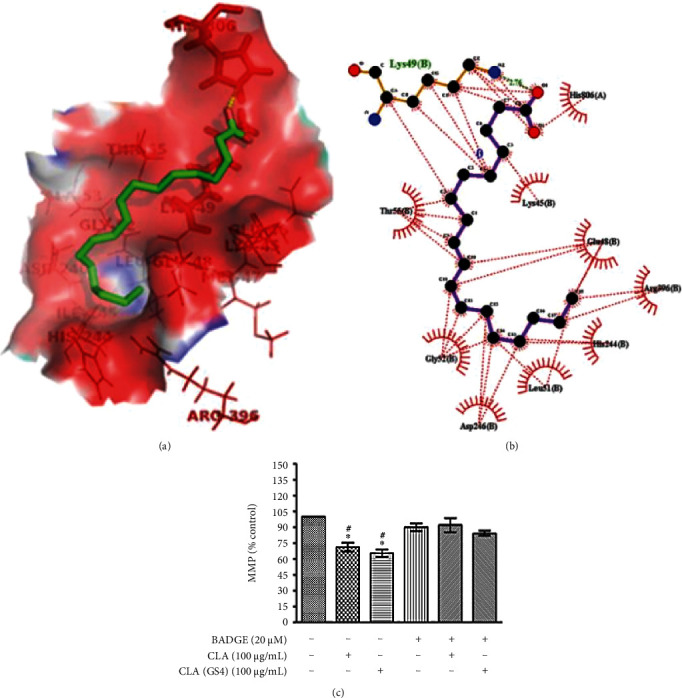
Effect of biohydrogenation-derived CLA on mitochondrial membrane potential (*Ψ*_m_): the hHK-II protein was retrieved from protein data bank and molecular docking was performed using AutoDock followed by LigPlot analysis. BE was used in order to rank structures in each cluster. (a) PyMOL view of molecular interaction between c9t11-CLA and hHK-II. (b) LigPlot analysis of c9t11-CLA with the amino acid residues of hHK-II. (c) The HCT-116 cells were pre-treated with BADGE followed by 100 *μ*g/mL CLA/CLA_GS4_ treatment for 24 hours subsequently Rh 123 staining. The fluorescence intensity was measured using fluorescence spectrophotometry and the data were expressed as % of control. Statistical analysis of data (means ± SE) was determined by one way-ANOVA with *post hoc* Newman–Keuls analysis and depicted as ∗*versus* control; #*versus* BADGE control.

Previous studies have shown that voltage dependent anionic channel (VDAC) can bind not only to the protein involved in mitochondrial functions and cellular metabolism but also with pro or anti-apoptotic proteins of the Bcl-2 family, which subsequently contributes towards regulation of mitochondrial membrane permeability [16, 17]. Interestingly, it was noticed that VDAC can also interact with hexokinase, a cytosolic cell metabolism protein, and, thereby, transference in the exposure of mitochondria to pro-apoptotic proteins [18]. The higher expression of HK-II in cancer cells enables them to escape from programmed cell death through competes with Bcl_2_ family proteins for binding to VDAC along with metabolic advantage. Thus, it was postulated that CLA can bind with HK-II, which may interfere HK-II interaction with VDAC, making latter available for pro-apoptotic Bax protein binding that may cause membrane depolarization. Molecular docking study showed that CLA can bind efficiently with HK-II (PDB ID: 2nzt), with BE −2.59 kcal/mol (Figure 9(a)). Furthermore, LigPlot analysis revealed that 9, 11-CLA forms hydrophobic interaction with active site residues of human HK-II protein, such as HIS806(A), LYS45(B), GLU18(B), ARG296(B), HIS244(B), LEU51(B), ASP246(B), GLY52(B), and THR56(B) (Figure 9(b)). Furthermore, the HK-II protein B-chain (amino acid residue LYS49) forms hydrogen bonding (H-bond) interaction (bond length = 2.76 Å) with the OH− of residue of 9, 11-CLA ligand, indicating the possible interaction of CLA with HK-II.

#### 3.8.2. Mitochondrial Membrane Depolarization

Previous study revealed that PPAR*γ* agonist can interfere MMP through the binding with HK-II, which in turn allows binding of pro-apoptotic Bad with VDAC in outer membrane of mitochondria inducing depolarization of mitochondrial membrane [19]. The molecular docking study revealed the binding between CLA and HK-II, suggesting its possible role in MMP depolarization (∆*ψ*_m_). Therefore, MMP was in HCT116 cells treated with CLA/CLA_GS4_ by quantification of Rh123 fluorescence intensity with fluorospectrometric. As shown in Figure 9(c), the CLA/CLA_GS4_ significantly (*P* ≤ 0.05) reduced membrane potential in CLA/CLA_GS4_ treated cancer cells, which was apparent from reduced fluorescent intensity, suggesting that cells were undergoing through ∆*ψ*_m_ (Figure 9(c)). Interestingly, the polarization of mitochondria was rescued partially by PPAR*γ*-antagonist indicating the PPAR*γ*-dependent mechanisms in apoptosis.

## 4. Discussion

Probiotic, which delivers the health beneficial effects of CLA at the host microbe interface and induces anti-proliferation in colonocytes, is an effective treatment for the mitigation of CC [9]. In this study, we delineated the underlying apoptotic consequences induced by probiotic GS4 during CC mitigation. We found that probiotic GS4 can induce PPAR*γ* expression, which subsequently arbitrate the proliferation of CC cells, and induced apoptosis. Mechanistically, we found that CLA binding to HK-II opens VDAC-II and subsequently promotes mitochondrial membrane pore transition that leads to cancer cell apoptosis. In addition, we found that up-regulation of PPAR*γ* modulates the Warburg effect and downregulates proliferative cell signalling events in cancer cells.

The nuclear receptor PPAR*γ*, a transcriptional master regulator of cell metabolism, inhibits the growth of several common cancers including CC [20, 21]. It is expected that probiotic GS4, which have biohydrogenation ability to produce CLA, would enable them to modulate cancer cell metabolism through the modulation of PPAR*γ* in terms of their antiproliferative efficiency. Thus, study was performed to elucidate the possible role of PPAR*γ* in probiotic GS4 mediated anti-CC effects. The current study indicates that probiotic-derived CLA had upregulation of PPAR*γ*, which had anti-proliferative efficacy in HCT-116 cells that are known to express wild type and functional PPAR*γ*, and its antiproliferative activities were found associated with apoptosis as shown by annexin V staining. These finding substantiates previous observations, demonstrating that probiotic-derived CLA stimulates apoptosis of human mammary tumors [22]. This efficacy seen in current study seems to be facilitated by PPAR*γ* transactivation, because BADGE treatment reversed anti-proliferative effects induced by the CLA. Furthermore, it was difficult to achieve complete blockage of PPAR*γ* expression because of the relative low affinity and solubility of BADGE [23].

Besides reducing the expression of the arachidonic acid metabolizing enzymes COX-2 and 5-LOX, CLA/CLA_GS4_ also suppressed PGE_2_ secretion. PPAR*γ* is a potential regulator of arachidonic acid metabolism; agonists for PPAR*γ* effectively down regulate COX-2 and 5-LOX, which is found useful in the treatment of inflammation associated disorders, for instance, inflammatory bowel disease [24]. The current observations are in line with previous study, where NSAID drug ciglitazone inhibited CC through the modulation of COX-2 in human CC HT-29 cells [25]. Furthermore, study also suggests that CLA mediated PPAR*γ* transactivation ultimately resulted into altered expression of down effector 5-LOX and COX-2 expressions [24, 26].

A considerable conceptual advance in understanding of metabolic alterations in cancer cells has been led to hypothesize the potential implication of PPAR*γ* ligand in metabolic pathways linking to cancer cell proliferations. The observations indicate that PPAR*γ* upregulation by CLA of probiotic GS4 causes alteration in cellular glucose metabolism, which was evident from reduced glucose uptake that resulted in reduced cellular NAD^+^ level leading to disturbance in redox state. Loss of redox status of cancer cells can be prevented by PPAR*γ* antagonist, suggesting PPAR*γ* vital role in this process. The most important revelation from the present study pertains to the linkage between metabolic alteration and cancer cell death. The study showed that perturbed glucose consumption and cellular NAD^+^ level by PPAR*γ* upregulation mediated by CLA effectively induced cell death. Previously, it was shown that PPAR-*γ* endogenous ligands, 2-cyano-3,12-dioxooleana-1,9-dien-28-oic acid and 15d-PGJ(2), can inhibit differentiation of myofibroblast by blocking phosphorylation of Akt (pAkt), and this mechanism was found to be PPAR-*γ*-independent [27]. Furthermore, the cell cycle arrest at G0/G1 stage confirms these observations. Earlier studies suggested that the PPAR*γ* mediated metabolic alterations can cause reactive oxygen species (ROS)-mediated retinoblastoma (Rb) hypophosphorylation affecting cell cycle progression [28]. The current study did not estimate the Rb phosphorylation, however it is apparent from the increased ROS level and cell cycle arrest, that these effects are reversed in presence of PPAR*γ* antagonist. It further suggests that PPAR*γ* transactivation might have induced ROS-mediated Rb hypophosphorylation followed by cell cycle arrest leading to cell death. The present study further corroborated the anti-proliferative efficacy of CLA against breast cancer MCF-7 cells [29].

Using FACS and spectrofluorometric analysis, it was found that biohydrogenation-derived CLA induced a marked decrease of MMP in association with increased annexin V/PI stained cells suggesting that a loss of MMP is an important mechanism by which CLA induced apoptosis, a notion further supported by observation that PPAR*γ* antagonist partly abolished loss of MMP. The MMP depolarization is physiological relevant based on our previous *in vivo* observation that probiotic GS4 intervention induced intrinsic apoptotic pathways in induced cancerous colonocytes in the experimental mice model [9]. Previously, PPAR*γ* agonist was observed to induce mitochondria mediated apoptosis in tumor cells and reduce MMP in cancer cells [28]. Interestingly, a marked decreased annexin V/PI stained cells/AI was observed in the presence of PPAR*γ* antagonist, indicating a possible PPAR*γ*-independent mechanism which might also serving as contributing factors in apoptosis stimulation. Previously, HK-II, which is highly expressed in many tumor cells [30], contributing towards cancer cells metabolic transformations commonly referred to as the Warburg effect [31]. The role of HK-II dependent cell death in cancerous cells is also prominent from examples of displacement from mitochondrial associated membranes resulting in a calcium dependent calpain activation leading to death. Research studies indicated that HK-II bind to outer mitochondrial membrane (OMM) through VDAC, also called as porin [32], a major contributor in the maintenance of membrane potential. The HK-II and VADC interaction has been reported to reconstitute lipid membrane and affecting its characteristics permeability for ions and; hence, promoting a closed state of the channel [33]. In the current study, molecular docking and LigPlot analysis revealed that HK-II carries a binding domain for c9, t11-CLA, and thus, it is proposed that this interaction that causes the conformational changes in HK-II leads to its dissociation from VDAC at OMM and ultimately causes mitochondrial membrane depolarization. Molecular docking and LigPlot observations that HK-II carries a binding domain for CLA, and mitochondrial membrane depolarization attest this hypothesis [11]. The current observations were found to be consistent with previous reports where CLA has direct impact over VDAC expression in skeletal muscles cells [34]. Moreover, the observation with membrane blabbing, chromatin condensation, and elevated AI/annexin V-PI stained cells and cell cycle arrest at sub-G0 stage in CLA/CLA_GS4_ treated cells further confirms the apoptosis in human CC cells.

Taken all together, it is inferred that mechanistically, the upregulation of PPAR*γ* by CLA producing probiotic *P*. *pentosaceus* GS4 can alter cancer cell metabolism in association with triggering apoptosis in CC. The findings from current study would pave the way for probiotic/its derived metabolites in CC and associated metabolic disorders.

## Data Availability

Data are available upon request to either Prof. Ghosh or Vinay Dubey.

## References

[1] Stewart B. W., Wild C. P. (2014). *World Cancer Report 2014*.

[2] Varga T., Czimmerer Z., Nagy L. (2011). PPARs are a unique set of fatty acid regulated transcription factors controlling both lipid metabolism and inflammation. *Biochimica et Biophysica Acta*.

[3] Nolte R. T., Wisely G. B., Westin S. (1998). Ligand binding and co-activator assembly of the peroxisome proliferator-activated receptor-gamma. *Nature*.

[4] Altiok S., Xu M., Spiegelman B. M. (1997). PPAR*γ* induces cell cycle withdrawal: Inhibition of E2f/DP DNA-binding activity via down-regulation of PP2A. *Genes and Development*.

[5] Chen F., Harrison L. E. (2005). Ciglitazone-induced cellular anti-proliferation increases p27 kip1 protein levels through both increased transcriptional activity and inhibition of proteasome degradation. *Cellular Signalling*.

[6] Chen F., Kim E., Wang C. C., Harrison L. E. (2005). Ciglitazone-induced p27 gene transcriptional activity is mediated through Sp1 and is negatively regulated by the MAPK signaling pathway. *Cellular Signalling*.

[7] Komatsu Y., Ito I., Wayama M. (2008). PPAR*γ* ligands suppress the feedback loop between E2F2 and cyclin-E1. *Biochemical and Biophysical Research Communications*.

[8] Bonofiglio D., Gabriele S., Aquila S. (2009). Peroxisome proliferator-activated receptor gamma activates fas ligand gene promoter inducing apoptosis in human breast cancer cells. *Breast Cancer Research and Treatment*.

[9] Dubey V., Ghosh A. R., Bishayee K., Khuda-Bukhsh A. R. (2016). Appraisal of the anti-cancer potential of probiotic *Pediococcus pentosaceus* GS4 against colon cancer: in vitro and in vivo approaches. *Journal of Functional Foods*.

[10] Ghersi D., Sanchez R. (2009). Improving accuracy and efficiency of blind protein-ligand docking by focusing on predicted binding sites. *Proteins: Structure, Function and Bioinformatics*.

[11] Muniyan R., Gurunathan J. (2016). Lauric acid and myristic acid from *Allium sativum* inhibit the growth of *Mycobacterium tuberculosis* H37Ra: in silico analysis reveals possible binding toprotein kinase B. *Pharmaceutical Biology*.

[12] Wallace A. C., Laskowski R. A., Thornton J. M. (1995). LIGPLOT—a program to generate schematic diagrams of protein ligand interactions. *Protein Engineering*.

[13] Liu K., Liu P. C., Liu R., Wu X. (2015). Dual AO/EB staining to detect apoptosis in osteosarcoma cells compared with flow cytometry. *Medical Science Monitor Basic Research*.

[14] Takeuchi M., Yamamoto T. (2015). Apoptosis induced by NAD depletion is inhibited by KN-93 in a CaMKII-independent manner. *Experimental Cell Research*.

[15] Cantó C., Menzies K. J., Auwerx J. (2015). NAD(+) metabolism and the control of energy homeostasis: a balancing act between mitochondria and the nucleus. *Cell Metabolism*.

[16] Tsujimoto Y., Shimizu S. (2002). The voltage-dependent anion channel: an essential player in apoptosis. *Biochimie*.

[17] Baines C. P., Kaiser R. A., Sheiko T., Craigen W. J., Molkentin J. D. (2007). Voltage-dependent anion channels are dispensable for mitochondrial-dependent cell death. *Nature Cell Biology*.

[18] Azoulay H., Israelson A., Abu H. S., Shoshan-Barmatz V. (2004). In self-defence: hexokinase promotes voltage-dependent anion channel closure and prevents mitochondria-mediated apoptotic cell death. *The Biochemical Journal*.

[19] Banerjee J., Ghosh S. (2004). Bax increases the pore size of rat brain mitochondrial voltage-dependent anion channel in the presence of tBid. *Biochemical and Biophysical Research Communications*.

[20] Kelly D., Campbell J. I., King T. P. (2004). Commensal anaerobic gut bacteria attenuate inflammation by regulating nuclear-cytoplasmic shuttling of PPAR-gamma and RelA. *Nature Immunology*.

[21] Chiang J. Y. L., Li T. (2009). Regulation of bile acid and cholesterol metabolism by PPARs. *PPAR Research*.

[22] Ou L., Ip C., Lisafeld B., Ip M. M. (2007). Conjugated linoleic acid induces apoptosis of murine mammary tumor cells via Bcl-2 loss. *Biochemical and Biophysical Research Communications*.

[23] Wright H. M., Clish C. B., Mikami T. (2000). A synthetic antagonist for the peroxisome proliferator-activated receptor gamma inhibits adipocyte differentiation. *The Journal of Biological Chemistry*.

[24] Ye Y. N., Wu W. K., Shin V. Y., Bruce I. C., Wong B. C., Cho C. H. (2005). Dual inhibition of 5-LOX and COX-2 suppresses colon cancer formation promoted by cigarette smoke. *Carcinogenesis*.

[25] Shiau C. W., Yang C. C., Kulp S. K. (2005). Thiazolidenediones mediate apoptosis in prostate cancer cells in part through inhibition of Bcl-xL/Bcl-2 functions independently of PPAR*γ*. *Cancer Research*.

[26] Ochoa J. J., Farquharson A. J., Grant I., Moffat L. E., Heys S. D., Wahle K. W. (2004). Conjugated linoleic acids (CLAs) decrease prostate cancer cell proliferation: different molecular mechanisms for cis-9, trans-11, and trans-10 cis-12 isomers. *Carcinogenesis*.

[27] Kulkarni A. A., Thatcher T. H., Olsen K. C., Maggirwar S. B., Phipps R. P., Sime P. J. (2011). PPAR-*γ* ligands repress TGF*β*-induced myofibroblast differentiation by targeting the PI3K/Akt pathway: Implications for therapy of fibrosis. *PLoS One*.

[28] Srivastava N., Kollipara R. K., Singh D. K. (2014). Inhibition of cancer cell proliferation by PPAR*γ* is mediated by a metabolic switch that increases reactive oxygen species levels. *Cell Metabolism*.

[29] Rakib M. A., Lee W. S., Kim G. S., Han J. H., Kim J. O., Ha Y. L. (2013). Antiproliferative action of conjugated linoleic acid on human MCF-7 breast cancer cells mediated by enhancement of Gap junctional intercellular communication through inactivation of NF-*κ*B. *Evidence-Based Complementary and Alternative Medicine*.

[30] Pastorino J. G., Hoek J. B. (2003). Hexokinase II: the integration of energy metabolism and control of apoptosis. *Current Medicinal Chemistry*.

[31] Palsson-Mcdermott E. M., O’Neill L. A. J. (2013). The Warburg effect then and now: from cancer to inflammatory diseases. *BioEssays*.

[32] Pastorino J. G., Hoek J. B. (2008). Regulation of hexokinase binding to VDAC. *Journal of Bioenergetics and Biomembranes*.

[33] Anflous K., Cai Z. J., Craigen W. J. (2007). VDAC1 serves as a mitochondrial binding site for hexokinase in oxidative muscles. *Biochimica et Biophysica Acta - Bioenergetics*.

[34] Kim Y., Park Y. (2015). Conjugated linoleic acid (CLA) stimulates mitochondrial biogenesis signaling by the upregulation of PPARgamma coactivator 1alpha (PGC-1alpha) in C2C12 cells. *Lipids*.

[35] Han S. W., Roman J. (2007). Peroxisome proliferator-activated receptor gamma: a novel target for cancer therapeutics?. *Anticancer Drugs*.

[36] Dubey V., Ghosh A. R., Mandal B. K. (2012). Appraisal of conjugated linoleic acid production by probiotic potential of Pediococcus spp. GS4. *Applied Biochemistry and Biotechnology*.

[37] Wang D. (2006). R N DuBois Prostaglandins and cancer. *Gut*.

